# Supplementary material for: Towards designing of a potential new HIV-1 protease inhibitor using QSAR study in combination with Molecular docking and Molecular dynamics simulations
Source: PLoS One. 2023 Apr 20;18(4):e0284539. doi: 10.1371/journal.pone.0284539 (PMC10118106; doi:10.1371/journal.pone.0284539)
Supplement: S1 File — (DOCX) [file pone.0284539.s001.docx]

**Towards designing of a potential new HIV-1 protease inhibitor using QSAR study in combination with Molecular Docking and Molecular Dynamics Simulations**

Mouna Baassi^1^, Mohamed Moussaoui^1^, Hatim Soufi^1^, Sanchaita Rajkhowa^2*^, Ashwani Sharma^3^, Subrata Sinha^2^ and Said Belaaouad^1^

^1^ Laboratory of Physical Chemistry of Materials, Faculty of Sciences Ben M’Sick, Hassan II University of Casablanca, BP7955 Sidi Othmane, Casablanca, Morocco.

^2^ Centre for Biotechnology and Bioinformatics, Dibrugarh University, Dibrugarh-786004, Assam, India.

^3^ CEO Insight BioSolutions, Pierre Joseph Colin 6 st, 35000 Rennes, France.

^*^Corresponding author: Dr. Sanchaita Rajkhowa

email id: s_rajkhowa@dibru.ac.in

**S1 Table: Molecular structure of the thirty-three compounds**

| Cpds | Structure | Cpds | Structure | Cpds | Structure | Cpds | Structure |
| --- | --- | --- | --- | --- | --- | --- | --- |
| 16a |  | **18c** |  | **17f** |  | **16i** |  |
| 17a |  | **16d** |  | **18f** |  | **17i** |  |
| 18a |  | **17d** |  | **16g** |  | **18i** |  |
| 16b |  | **18d** |  | **17g** |  | **16j** |  |
| 17b |  | **16e** |  | **18g** |  | **17j** |  |
| 18b |  | **17e** |  | **16h** |  | **18j** |  |
| 16c |  | **18e** |  | **17h** |  | **16k** |  |
| 17c |  | **16f** |  | **18h** |  | **17k** |  |

**18k**

**S2 Table: The descriptors computed using the following software (Gaussian, Chem3D, ChemSketch and MarvinSketch)**

| Gaussian | E_LUMO_(ev), E_HOMO_(ev), Potentiel chimique μ (eV), Dureté η (eV), Electronégativité χ(eV), Indice d'éléctrophilicité ω (eV), Molesse S, Energie totale (ev), Moment diplolaire μ, E_Gap_. |
| --- | --- |
| Chem3D | Henry's Law Constant, Mol Weight, Number of HBond Donors, Number of HBond Acceptors, Mol Refractivity, Partition Coefficient, LogP, LogS, Molecular Topological Index, Cluster Count, Balaban Index, Num Rotatable Bonds, Polar Surface Area ( Angstroms Squared), Radius, Shape Attribute, Shape Coefficient, Sum Of Degrees, Sum Of Valence Degrees, Topological Diameter, Wiener Index. |
| ChemSketch | %C, %H, %N, %O, %S, Molar Refractivity, Molar Volume, Parachor, Index of Refraction, Surface Tension, Density, Polarizability, Average Mass. |
| MarvinSketch | Molecular weight, Atom count, log P, Chemaxon HLB , Aliphatic atom count, Aliphatic bond count , Atom count, Bond count, Chain atom count, Chain bond count, Rotatable bond count, Platt index, Randic index, Balaban index, Harary index, Hyper wiener index, Szeged index, Wiener index, Wiener polarity, Dreiding energy, MMFF94 energy, Minimal projection area, Maximal projection area, Minimal projection radius, Maximal projection radius, Length perpendicular to the max area, Length perpendicular to the min area, van der Waals volume, Polar surface area, Van der Waals surface area (3D), Donor count, Donor sites, Acceptor count, Acceptor sites, Refractivity. |

**S3 Table: Y-randomization results for the generated model**

| **Rand** | **R_Rand_** | **R^2^_Rand_** | **Q^2^_cv (Rand)_** | **Rand** | **R_Rand_** | **R^2^_Rand_** | **Q^2^_cv (Rand)_** | **Rand** | **R_Rand_** | **R^2^_Rand_** | **Q^2^_cv (Rand)_** | **Rand** | **R_Rand_** | **R^2^_Rand_** | **Q^2^_cv (Rand)_** |
| --- | --- | --- | --- | --- | --- | --- | --- | --- | --- | --- | --- | --- | --- | --- | --- |
| **1** | 0.39 | 0.15 | -0.28 | **26** | 0.34 | 0.12 | -0.31 | **51** | 0.26 | 0.07 | -0.44 | **76** | 0.42 | 0.18 | -0.22 |
| **2** | 0.42 | 0.18 | -0.20 | **27** | 0.45 | 0.20 | -0.21 | **52** | 0.33 | 0.11 | -0.33 | **77** | 0.59 | 0.35 | 0.03 |
| **3** | 0.20 | 0.04 | -0.48 | **28** | 0.38 | 0.14 | -0.29 | **53** | 0.25 | 0.06 | -0.41 | **78** | 0.42 | 0.18 | -0.19 |
| **4** | 0.18 | 0.03 | -0.38 | **29** | 0.15 | 0.02 | -0.51 | **54** | 0.49 | 0.24 | -0.09 | **79** | 0.24 | 0.06 | -0.41 |
| **5** | 0.37 | 0.14 | -0.32 | **30** | 0.35 | 0.13 | -0.31 | **55** | 0.17 | 0.03 | -0.50 | **80** | 0.14 | 0.02 | -0.46 |
| **6** | 0.08 | 0.01 | -0.52 | **31** | 0.68 | 0.46 | 0.21 | **56** | 0.27 | 0.07 | -0.46 | **81** | 0.26 | 0.07 | -0.29 |
| **7** | 0.13 | 0.02 | -0.40 | **32** | 0.33 | 0.11 | -0.34 | **57** | 0.32 | 0.10 | -0.32 | **82** | 0.32 | 0.10 | -0.29 |
| **8** | 0.28 | 0.08 | -0.35 | **33** | 0.30 | 0.09 | -0.45 | **58** | 0.43 | 0.18 | -0.15 | **83** | 0.32 | 0.10 | -0.34 |
| **9** | 0.41 | 0.17 | -0.24 | **34** | 0.48 | 0.24 | -0.14 | **59** | 0.29 | 0.08 | -0.37 | **84** | 0.30 | 0.09 | -0.32 |
| **10** | 0.50 | 0.25 | -0.10 | **35** | 0.39 | 0.16 | -0.28 | **60** | 0.36 | 0.13 | -0.38 | **85** | 0.46 | 0.21 | -0.10 |
| **11** | 0.57 | 0.32 | 0.03 | **36** | 0.57 | 0.32 | 0.01 | **61** | 0.34 | 0.12 | -0.31 | **86** | 0.19 | 0.04 | -0.37 |
| **12** | 0.45 | 0.20 | -0.14 | **37** | 0.30 | 0.09 | -0.37 | **62** | 0.36 | 0.13 | -0.34 | **87** | 0.23 | 0.05 | -0.44 |
| **13** | 0.37 | 0.14 | -0.30 | **38** | 0.50 | 0.25 | -0.22 | **63** | 0.30 | 0.09 | -0.38 | **88** | 0.35 | 0.12 | -0.32 |
| **14** | 0.22 | 0.05 | -0.41 | **39** | 0.19 | 0.04 | -0.45 | **64** | 0.44 | 0.20 | -0.26 | **89** | 0.46 | 0.21 | -0.09 |
| **15** | 0.42 | 0.17 | -0.27 | **40** | 0.41 | 0.17 | -0.28 | **65** | 0.26 | 0.07 | -0.39 | **90** | 0.35 | 0.12 | -0.37 |
| **16** | 0.26 | 0.07 | -0.40 | **41** | 0.10 | 0.01 | -0.52 | **66** | 0.35 | 0.12 | -0.22 | **91** | 0.26 | 0.07 | -0.36 |
| **17** | 0.48 | 0.23 | -0.18 | **42** | 0.58 | 0.33 | -0.03 | **67** | 0.49 | 0.24 | -0.14 | **92** | 0.43 | 0.18 | -0.19 |
| **18** | 0.08 | 0.01 | -0.47 | **43** | 0.57 | 0.33 | -0.05 | **68** | 0.59 | 0.35 | 0.00 | **93** | 0.49 | 0.24 | -0.10 |
| **19** | 0.33 | 0.11 | -0.29 | **44** | 0.27 | 0.07 | -0.37 | **69** | 0.31 | 0.10 | -0.36 | **94** | 0.60 | 0.35 | 0.05 |
| **20** | 0.39 | 0.15 | -0.24 | **45** | 0.39 | 0.15 | -0.29 | **70** | 0.39 | 0.15 | -0.21 | **95** | 0.40 | 0.16 | -0.32 |
| **21** | 0.10 | 0.01 | -0.43 | **46** | 0.34 | 0.12 | -0.36 | **71** | 0.37 | 0.13 | -0.28 | **96** | 0.23 | 0.05 | -0.34 |
| **22** | 0.31 | 0.10 | -0.36 | **47** | 0.23 | 0.05 | -0.48 | **72** | 0.46 | 0.21 | -0.14 | **97** | 0.25 | 0.06 | -0.45 |
| **23** | 0.30 | 0.09 | -0.32 | **48** | 0.22 | 0.05 | -0.53 | **73** | 0.51 | 0.26 | -0.13 | **98** | 0.33 | 0.11 | -0.31 |
| **24** | 0.42 | 0.17 | -0.28 | **49** | 0.34 | 0.12 | -0.29 | **74** | 0.33 | 0.11 | -0.34 | **99** | 0.28 | 0.08 | -0.48 |
| **25** | 0.47 | 0.22 | -0.13 | **50** | 0.42 | 0.18 | -0.15 | **75** | 0.14 | 0.02 | -0.50 | **100** | 0.62 | 0.38 | 0.01 |

| **Random Models’ Parameters** | **Average R_Rand_** | **Average R^2^_Rand_** | **Average Q^2^_cv (Rand)_** | **cRp^2^** |
| --- | --- | --- | --- | --- |
|  | 0.35 | 0.14 | -0.29 | 0.60 |

**S4 Table: Drug candidates’ descriptors and their predicted biological activity as well as the leverage values**

| **N** | **E_Gap_** | **HLC** | **PSA** | **DE** | **Pred(pIC50)** | **hi** |
| --- | --- | --- | --- | --- | --- | --- |
| **1** | 4.61 | 11.42 | 147.26 | 136.10 | 1.94 | 1.22 |
| **2** | 4.61 | 11.42 | 127.03 | 133.97 | 2.63 | 1.24 |
| **3** | 4.65 | 11.42 | 127.03 | 184.38 | 1.86 | 0.51 |
| **4** | 4.62 | 11.42 | 127.03 | 180.02 | 1.94 | 0.56 |
| **5** | 5.27 | 11.80 | 136.26 | 140.51 | 2.33 | 1.26 |
| **6** | 5.27 | 11.80 | 156.49 | 142.72 | 1.63 | 1.44 |
| **7** | 5.28 | 11.80 | 136.26 | 187.75 | 1.62 | 0.51 |
| **8** | 5.22 | 12.05 | 167.49 | 136.45 | 1.72 | 1.81 |
| **9** | 5.21 | 12.05 | 147.26 | 131.48 | 2.45 | 1.61 |
| **10** | 4.95 | 11.61 | 127.03 | 137.05 | 2.63 | 1.24 |
| **11** | 4.96 | 11.61 | 147.26 | 138.03 | 1.95 | 1.29 |
| **12** | 5.02 | 11.61 | 127.03 | 186.78 | 1.84 | 0.51 |
| **13** | 4.99 | 11.61 | 127.03 | 185.72 | 1.88 | 0.52 |
| **14** | 5.23 | 12.05 | 147.26 | 180.85 | 1.71 | 0.67 |
| **15** | 5.31 | 12.18 | 153.05 | 185.04 | 1.58 | 0.68 |
| **16** | **4.82** | **11.29** | **127.03** | **185.41** | **1.58** | **0.43** |
| **17** | 4.99 | 11.61 | 127.03 | 152.40 | 2.38 | 0.96 |
| **18** | 4.98 | 11.61 | 127.03 | 152.61 | 2.38 | 0.96 |
| **19** | 4.98 | 11.61 | 127.03 | 140.37 | 2.56 | 1.18 |
| **20** | 4.97 | 11.61 | 127.03 | 139.21 | 2.58 | 1.20 |
| **21** | 5.00 | 11.61 | 127.03 | 152.63 | 2.37 | 0.96 |
| **22** | 5.00 | 11.61 | 127.03 | 186.35 | 1.86 | 0.51 |
| **23** | 5.03 | 11.61 | 127.03 | 187.20 | 1.84 | 0.50 |
| **24** | 5.01 | 11.61 | 127.03 | 152.70 | 2.36 | 0.96 |

#### S5 Table: Non-bonded interactions between the binding site residues of the complex compounds from molecular docking analysis

| **Protein** | **Ligand** | **Hydrogen bonds interactions** | | **hydrophobic interactions** | **VDW** |
| --- | --- | --- | --- | --- | --- |
| **WT** | **DRV** | A | Ala28, Asp25 | Pro81, Ile50, Ala28, Ile47 | Ile84, Asp30, Gly84, Gly27, Gly49, Leu23, Val82 |
|  |  | B | Asp30, Gly27 | Ile50, Ile84, Val82, Ala28, Ile47 | Lys45, Leu76, Asp29, Leu23, Arg8, Pro81, Asp25, Gly49, Gly48 |
|  | **ND** | A | Ala28, Gly49, Gly27 | Ile84, Ala28, Asp29, Ile50, Pro81, Val82, Ile84, Ala28, Ile47 | Asp25, Leu23, Gly48, Val32, Asp30, Asp29 |
|  |  | B | Asp25 | Ile47, Pro81, Ala28, | Ile84, Gly48, Asp29, Asp30, Val32, Gly49, Gly27, Ile50, Leu23, Val82 |
| **MT** | **DRV** | A | Asp30, Asp25 | Ile84, Ile50, Pro81 | Ile32, Ala28, Val47, Leu76, Asp29, Gly48, Gly49, Gly27, THR80, Leu82, Leu23 |
|  |  | B | Gly48 | Ala28, Val47, Ile84, Ile32, Ile50 | Gly49, Asp25, Arg8, Leu82, Thr80, Pro81, Gly27, Leu76, Asp29, Asp30 |
|  | **ND** | A | Arg8, Gly49, Gly27 | Ile84, Ile50, Pro81, Ala28 | Gly48, Val47, Asp29, Asp25, Thr80,Leu82, Leu23 |
|  |  | B | Ala28, Asp25 | Ile84, Ala28, Ile50 | Leu23, Thr80, Pro81, Leu82, Arg8, Asp30, Asp29, Gly48, Gly49, Gly27 |

## **Mutations occurring in the mutant type protease 3TTP**

#### Multiple mutations are present in the multidrug resistant 3TTP as compared to the wild type, notably V13, R20, I32, F33, D35, I36, K41, T43, V47, M54, V62, V63, V71, T72, S73, P74, L82, V89 and L93 (S6 Table).

#### S6 Table: The sequence of the wild type protease and its alignment with the wild type sequence. The major DRV resistance-associated mutations are mentioned in green, minor mutations in blue and other mutations are colored in red [1]

| WT | PQITLWQRPLVT IKIGGQLKEALLDTGADDTVLEEMNLPGRWKPKMI GGIGGF I KVRQYDQI L IEICGHKAIGTVLVGPTPVNIIGRNLLTQ IGCTLNF |
| --- | --- |
| MT | PQITLWQRPLVTVKIGGQLREALLDTGADDTI FED I NLPGKWTPKMVGGIGGFMKVRQYDQVVIEICGHK VTSPVLVGPTPLNIIGRNVLTQLGCTLNF |

#### In the one hand, as shown in the table below (S7 Table), there is a major DRV resistance-associated mutation Val47 included in the active site of 3TTP while docked to Darunavir (S1 Fig), in the other hand, the active site of 3TTP while docked to the new drug is free of any possible mutation (S2 Fig), supporting the previous results (docking study) disclosing the affinity values of MT-DRV and MT-ND; -9.9 and -10.4 Kcal/mol respectively, we can conclude that effectively the complex compound MT-ND is showing higher stability as compared to MT-DRV.

#### S7 Table: The complex compounds’ (MT-DRV & MT-ND) active site residues

| MT-DRV | Chain A: Asp25, Asp30, Ile50, Pro81 and Ile84.Chain B: Ala28, Ile32, Val47, Gly48, Ile50 and Ile84. |
| --- | --- |
| MT-ND | Chain A: Arg8, Gly27, Ala28, Gly49, Ile50, Pro81 and Ile84.Chain B: Asp25, Ala28, Ile50 and Ile84. |

**S8 Table: Atoms from both; ligands and residues interacting with each other to form hydrogen bonds**

| **Protease** | **Ligand** | **Chain** | **Hydrogen Bonds** | **Distance** |
| --- | --- | --- | --- | --- |
| **WT** | **DRV** | **Chain A** | Ala28:HN :UNN0: O  Asp25:OD2 :UNN0: H | 2.97926  2.66083 |
|  |  | **Chain B** | Asp30:OD2 :UNN0: H  Gly27:O :UNN0: C | 2.18525  3.63548 |
|  | **ND** | **Chain A** | Ala28:HN :UNK0: O  Gly49:CA :UNK0: O  Gly27:O :UNK0: C | 2.53230  2.72055  3.16318 |
|  |  | **Chain B** | Asp25:OD2 :UNK0: H | 2.23578 |
| **MT** | **DRV** | **Chain A** | Asp30:O :UNN0: H  Asp30:OD2 :UNN0: H  Asp25:OD2 :UNN0: O | 2.79185  2.63386  3.02792 |
|  |  | **Chain B** | Gly48:O :UNN0:C | 3.40266 |
|  | **ND** | **Chain A** | Arg8:HH2 :UNK0:N  Gly49: CA: UNK0: O  Gly27:O :UNK0:C | 2.70719  3.50982  3.22732 |
|  |  | **Chain B** | Ala28: HN: UNK0: O  Asp25:OD2 :UNK0:H | 3.00662  2.21072 |

####
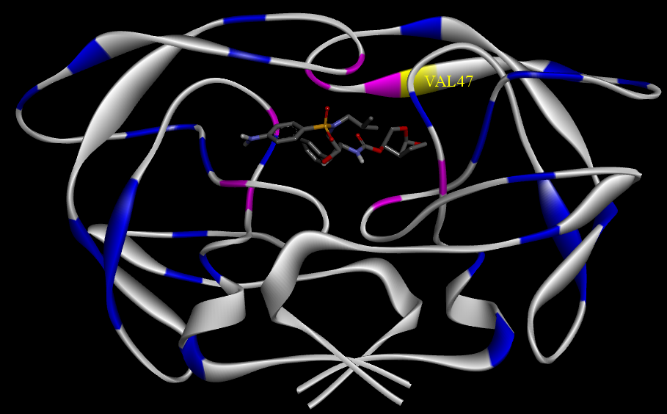


**S1 Figure: 3D-Structure of MT-DRV, mutations are colored in blue, the active site residues in purple and the mutation Val47 in yellow (a residue present in the active site)**

**
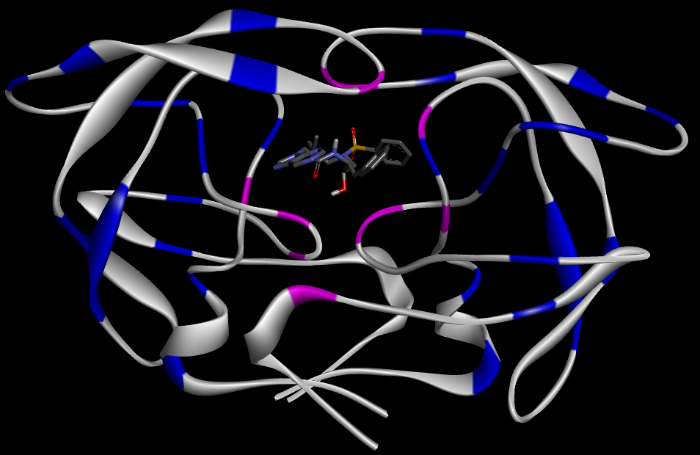
**

**S2 Figure: 3D-Structure of MT-ND, mutations are colored in blue and the active site residues in purple**

|  |  |  |  |  |
| --- | --- | --- | --- | --- |
|  |  |  |  |  |
|  |  |  |  |  |
|  |  |  |  |  |
|  |  |  |  |  |
|  |  |  |  |  |
|  |  |  |  |  |
|  |  |  |  |  |
|  |  |  |  |  |

# **References**

#### [1] Johnson VA, Calvez V, Gunthard HF, Paredes R, Pillay D, Shafer RW et al. (2013) Update of the drug resistance mutations in HIV-1: March 2013. Top Antivir Med 21, 6–14.
