# Supplementary figures and images for: Towards designing of a potential new HIV-1 protease inhibitor using QSAR study in combination with Molecular docking and Molecular dynamics simulations
Source: PLoS One. 2023 Apr 20;18(4):e0284539. doi: 10.1371/journal.pone.0284539 (PMC10118106; doi:10.1371/journal.pone.0284539)

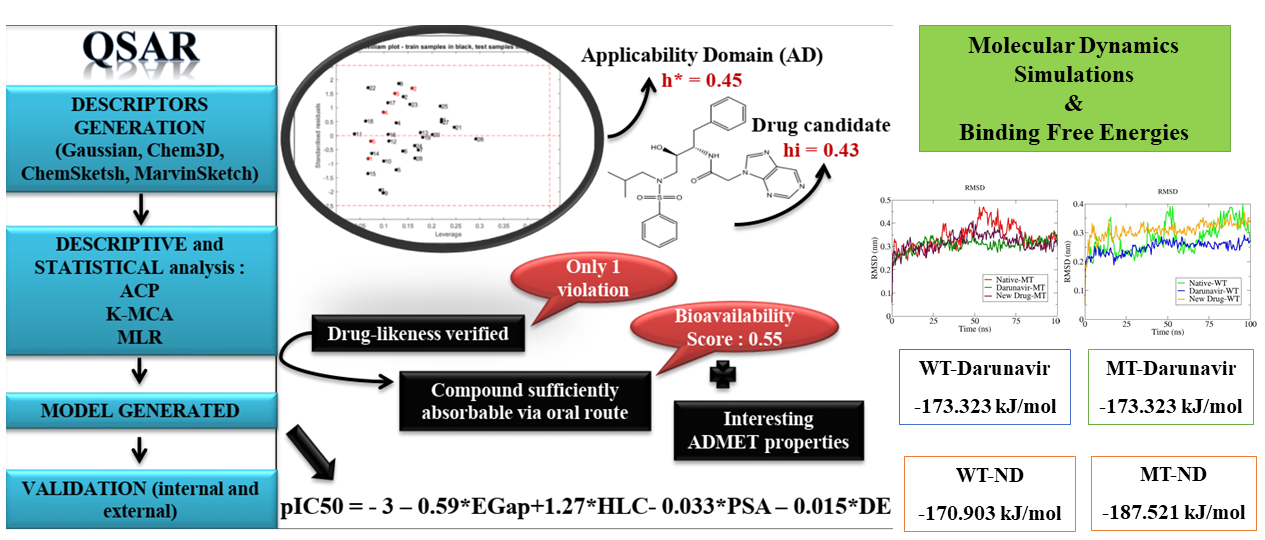

Supplement: S1 Graphical abstract — (TIF) [file pone.0284539.s002.tif]
